# Development of a syngeneic mouse model of epithelial ovarian cancer

**DOI:** 10.1186/1757-2215-3-24

**Published:** 2010-10-19

**Authors:** Bridget A Quinn, Fang Xiao, Laura Bickel, Lainie Martin, Xiang Hua, Andres Klein-Szanto, Denise C Connolly

**Affiliations:** 1 Women's Cancer Program, Fox Chase Cancer Center, 333 Cottman Avenue, Philadelphia, PA 19111-2497, USA; 2 Transgenic Facility Fox Chase Cancer Center, 333 Cottman Avenue, Philadelphia, PA 19111-2497, USA; 3 Department of Pathology, Fox Chase Cancer Center, 333 Cottman Avenue, Philadelphia, PA 19111-2497, USA; 4 Cancer Biology Program, Fox Chase Cancer Center, 333 Cottman Avenue, Philadelphia, PA 19111-2497, USA; 5 Department of Human and Molecular Genetics Virginia Commonwealth University School of Medicine 1220 E. Broad Street Room 7003 Richmond, VA 23298, USA

## Abstract

**Background:**

Most cases of ovarian cancer are epithelial in origin and diagnosed at advanced stage when the cancer is widely disseminated in the peritoneal cavity. The objective of this study was to establish an immunocompetent syngeneic mouse model of disseminated epithelial ovarian cancer (EOC) to facilitate laboratory-based studies of ovarian tumor biology and preclinical therapeutic strategies.

**Methods:**

Individual lines of Tg*MISIIR-TAg *transgenic mice were phenotypically characterized and backcrossed to inbred C57BL/6 mice. In addition to a previously described line of EOC-prone mice, two lines (Tg*MISIIR-TAg-Low*) were isolated that express the oncogenic transgene, but have little or no susceptibility to tumor development. Independent murine ovarian carcinoma (MOVCAR) cell lines were established from the ascites of tumor-bearing C57BL/6 Tg*MISIIR-TAg *transgenic mice, characterized and tested for engraftment in the following recipient mice: 1) severe immunocompromised immunodeficient (SCID), 2) wild type C57BL/6, 3) oophorectomized tumor-prone C57BL/6 Tg*MISIIR-TAg *transgenic and 4) non-tumor prone C57BL/6 Tg*MISIIR-TAg-Low *transgenic. Lastly, MOVCAR cells transduced with a luciferase reporter were implanted in Tg*MISIIR-TAg-Low *mice and *in vivo *tumor growth monitored by non-invasive optical imaging.

**Results:**

Engraftment of MOVCAR cells by i.p. injection resulted in the development of disseminated peritoneal carcinomatosis in SCID, but not wild type C57BL/6 mice. Oophorectomized tumor-prone Tg*MISIIR-TAg *mice developed peritoneal carcinomas with high frequency, rendering them unsuitable as allograft recipients. Orthotopic or pseudo-orthotopic implantation of MOVCAR cells in Tg*MISIIR-TAg-Low *mice resulted in the development of disseminated peritoneal tumors, frequently accompanied by the production of malignant ascites. Tumors arising in the engrafted mice bore histopathological resemblance to human high-grade serous EOC and exhibited a similar pattern of peritoneal disease spread.

**Conclusions:**

A syngeneic mouse model of human EOC was created by pseudo-orthotopic and orthotopic implantation of MOVCAR cells in a susceptible inbred transgenic host. This immunocompetent syngeneic mouse model presents a flexible system that can be used to study the consequences of altered gene expression (e.g., by ectopic expression or RNA interference strategies) in an established MOVCAR tumor cell line within the ovarian tumor microenvironment and for the development and analysis of preclinical therapeutic agents including EOC vaccines and immunotherapeutic agents.

## Background

Ovarian cancer is the most common cause of death from gynecologic malignancies and the fifth most common cause of cancer death in women in the United States [1]. Ovarian adenocarcinomas account for 85-90% of all cancers of the ovary. The initiating cell population for EOC remains to be exactly defined, with different evidence suggesting tumors originate from the ovarian surface epithelium (OSE), inclusion cysts lined by OSE [2-5] or alternatively, the fallopian tube epithelium [6] or components of the secondary Müllerian system, including the epithelial cells of the *rete ovarii*, paraovarian/paratubal cysts, endosalpingiosis, endometriosis or endomucinosis [7]. The lack of clarity regarding tumor origin stems from the fact that unlike epithelial cancers arising in other organs, a well-defined disease spectrum consisting of benign, invasive and metastatic lesions has not been identified for EOC. This is due at least in part to that fact that the majority of cases are identified at advanced stage when disease has spread beyond the ovary. Another reason is the morphologic complexity of common EOCs which consist of several distinct histologic subtypes; these include serous, endometrioid, mucinous and clear cell cancers.

Progress in ovarian cancer research has been slowed by the lack of suitable animal models that exhibit features of human disease. Genetically manipulable mammalian models of spontaneous ovarian cancer are rare, particularly those representing ovarian adenocarcinomas. Human and rodent models of spontaneous *ex vivo *transformation of OSE have been described [8-10]. One of these models, a syngeneic mouse model of EOC [10], has been extensively used for preclinical studies of therapeutic agents and studies of the tumor microenvironment [11-18]. Early attempts to produce murine EOC models using transgenic or other genetic engineering approaches resulted in the development of granulosa cell tumors [19-24]. More recently, a number of laboratories have developed genetically engineered mouse (GEM) models of EOC by using *ex vivo *transformation [25,26], transgenic [27,28] and conditional gene expression strategies [29-31]. To date, due to the lack of a suitable GEM model expressing Cre-recombinase, the strategy most frequently employed for conditional gene expression in the ovarian epithelium involves survival surgery for intrabursal injection of recombinant Adenovirus-Cre [29-34].

Recently, our group developed a spontaneous transgenic mouse model of EOC by expressing the oncogenic early region of SV40 under the transcriptional control of the Müllerian inhibiting substance type II receptor gene promoter [27,28]. Although SV40 TAg expression is not directly associated with the development of human cancer, its expression results in functional inactivation of the critical tumor suppressors p53 and Rb. Mutation of *TP53 *is, by far, the most common genetic alteration observed in EOC, particularly the serous subtype [35,36]. Direct mutation or loss of *Rb *or its downstream signaling mediators are also common in EOCs [37-41]. Via binding and inhibition of PP2A, SV40 tag also results in activation of PI3K/AKT and mitogen activated protein kinase (MAPK) signaling [42], pathways frequently activated in human EOC [43]. A stable transgenic line of Tg*MISIIR-TAg *mice was established in which female mice develop bilateral ovarian carcinoma with 100% penetrance [28]. To date, this is the only GEM model that develops spontaneous EOC with pathological features of serous EOC that does not require extensive surgical manipulation to induce the phenotype. Like human EOC, female Tg*MISIIR-TAg *mice with significant tumor burden exhibit no apparent symptoms of illness and disease dissemination is typically restricted to the peritoneum [27,28]. Murine ovarian carcinoma (MOVCAR) cell lines isolated from the ascites and primary tumors of these mice share many molecular features with human tumors [27,28,44-48] and are well suited to experimental analysis *in vitro*. With these reagents, the expression levels of specific genes can be experimentally manipulated and properties of MOVCAR cell lines can be assessed *in vitro*. However, the lack of a syngeneic recipient for manipulated MOVCAR cells has limited the analysis of the *in vivo *effects of genetic alterations in the model to studies in immunodeficient mice. The present study describes the identification of non-tumor prone lines of Tg*MISIIR-TAg *transgenic mice that can be used as syngeneic recipients for MOVCAR cell allografts. The availability of this syngeneic model affords the opportunity to study the *in vivo *effects of genetic alterations on tumor properties and on interactions between tumor cells and their microenvironment in an immunocompetent host. Moreover, this immunocompetent mouse model of EOC is suitable for studies of immune-based therapeutic strategies and vaccine development.

## Methods

### Transgenic mice and backcrosses

All procedures involving mice were approved by the Fox Chase Cancer Center (FCCC) Institutional Animal Care and Use Committee (IACUC) and all mice were maintained under specific pathogen free conditions. Individual transgenic Tg*MISIIR-TAg *founder mice were generated in the FCCC Transgenic Facility in a first generation hybrid genetic background of C57BL/6 and C3H (B6C3F1) and genotyped by PCR amplification as previously described [27]. Transgenic founders were crossed with wild type C57BL/6 mice (obtained from the FCCC Laboratory Animal Facility) to establish breeding lines. Relevant lines of EOC-prone and non-tumor-prone Tg*MISIIR-TAg *mice were maintained as hemizygotes and backcrossed for a minimum of ten generations to wild type C57BL/6 mice to generate genetically pure lines of C57BL/6 Tg*MISIIR-TAg *mice.

### Cell lines and culture conditions

Pure C57BL/6 MOVCAR cell lines, including MOVCAR 12, 5009 [49], 5025, 5183, 5438, 5447 and 5612, were established from bulk ascites isolated from individual ovarian tumor-bearing C57BL/6 Tg*MISIIR-TAg *mice as previously described [27]. Tumorigenic spontaneously transformed murine ovarian surface epithelial cell (MOSEC) lines ID-8, IF-5 and IG-10 were a gift from Dr. Katherine Roby, University of Kansas Medical Center, and ID-8 cells stably overexpressing murine VEGF164 were a gift from Dr. George Coukos, University of Pennsylvania. All MOVCAR and MOSEC cells were maintained in DMEM supplemented with 4% FBS, 1× Insulin/Transferrin/Selenium-A (ITS, supplied as 100× stock from Gibco/Invitrogen), penicillin/streptomycin (100 units/mL and 100 μg/mL, respectively) and 2 mM l-glutamine and incubated at 37°C in 5% CO_2_. Culture medium was changed once weekly and cells were trypsinized and passaged at 4-5 day intervals when they reached confluence. MOVCAR cells were prepared for *in vivo *injection as described [49]. For *in vivo *imaging, cells were transduced with a retroviral construct encoding the firefly luciferase gene (pWZL-Luc, generously provided by Dr. Maureen Murphy, FCCC) using standard methods.

### Immunoblot and immunoprecipitation

To prepare lysates for immunoblot analysis, cells were washed with cold PBS, lysed with M-PER mammalian protein extraction reagent (Thermo Scientific, Rockford, IL) supplemented with a cocktail of protease inhibitors (Complete Mini, Roche, Indianapolis, IN) and protein concentration was determined by BCA method (Thermo Scientific, Rockford, IL). Equal amounts of protein samples were resolved by SDS-PAGE gel electrophoresis on 12% acrylamide gels and transferred to polyvinylidene difluoride membrane (Immobilon, Millipore Corp., Bedford, MA). Membranes were blocked in 5% milk and 0.1% Tween-20 in 1× PBS for 1 h prior to incubation with primary antibodies recognizing SV40 TAg (Pab 101) and mouse p53 (Pab 240) obtained from Santa Cruz Biotechnology, Inc. at 1:1000 dilution. Horseradish peroxidase-conjugated secondary antibodies were used according to manufacturer's protocols. Immunoreactivity was visualized using the ECL system and was exposed to BioMax MR film (Eastman Kodak Co.).

For immunoprecipitation, cells were grown in 100-mm plates and lysed in 1 ml M-PER mammalian protein extraction reagent. The whole cell lysates were incubated with SV40 TAg antibody (Pab101) at a dilution of 1:100 at 4°C overnight with constant mixing. Protein A beads (40 μl) were added and mixed for 3 h at 4°C. Immunoprecipitates were then washed 5 times with M-PER mammalian protein extraction reagent and pellets resuspended in Laemli buffer for protein electrophoresis and immunoblot blot analysis performed as described above with antibodies against TAg and p53.

### Cell cycle analysis

Cells were prepared for cell cycle analysis using the fluorescent nuclear stain propidium iodide and fluorescent sorting was carried out using the Guava Personal Cell Analysis machine exactly as described by the manufacturer (Guava Technologies).

### RNA preparation, quantitative reverse transcription PCR

Total RNA was isolated from MOVCAR cells using the RNA Easy Mini Kit (Qiagen). With the assistance of the FCCC Genomics Facility, levels of *Mdm2 *mRNA expression were evaluated by real-time quantitative reverse transcription PCR (qRT-PCR) using Taqman technology with probe sets for *Mdm2 *and *Hprt1 *obtained from Applied Biosystems, Carlsbad, CA.

### Quantitation of secreted VEGF by ELISA

Cells (5 × 10^5^) were plated in triplicate in 6-well dishes and grown in complete medium for 72 hours. The conditioned culture medium was removed and the level of secreted VEGF present in the medium was determined by ELISA using the Mouse VEGF Quantikine Elisa Kit (R&D systems, Minneapolis, MN). After removal of the conditioned culture supernatant, cells were immediately rinsed with PBS, trypsinized and the number of cells present in each well was counted. Secreted VEGF levels were normalized to the total number of cells present in the sample to determine the amount of VEGF/10^4 ^cells. Three independent assays were performed and the amount of secreted VEGF/10^4 ^cells expressed as the mean value for each cell line tested.

### Oophorectomy and MOVCAR cell allografts

Four to six week-old ovarian tumor-prone Tg*MISIIR-TAg *mice were anesthetized by i.p. injection of 95 μl per 10 gram body weight of 10 mg/mL Ketamine hydrochloride and 1 mg/mL Xylazine hydrochloride in sterile saline and subjected to oophorectomy using a standard asceptic surgical procedure commonly used for transgenic embryo injection to expose the ovarian fat pad and ovary (described in detail in [49]). Once exposed, a small incision was made in the ovarian bursa that enabled removal of the resident ovary and/or fallopian tube. The ovarian bursa was sealed with surgical glue and the reproductive tract returned through the incision in the body wall. The surgical incision was closed with wound clips. The same surgical procedure was used for orthotopic (i.b.) injection of MOVCAR cells into recipient mice. Methods for i.b. and i.p. (pseudo-orthotopic) injections of MOVCAR cells were previously described in detail [49].

### Preparation and analysis of tissues, histology and immunohistochemistry

All mice were euthanized by CO_2 _asphyxiation, necropsied and examined for gross abnormalities. Pathologically altered organs, entire reproductive tracts and representative specimens of multiple organs and tissues, including the brain, lung, liver, kidney, spleen, pancreas and intestine were removed at necropsy, fixed in 10% (v/v) neutral buffered formalin (NBF) overnight, transferred to 70% ethanol and paraffin-embedded. In mice with evident tumor, specimens of the tumor tissue were also excised, snap frozen in liquid N_2 _and stored at -80°C. For histological analysis, 5 μm formalin fixed paraffin embedded tissue sections were cut for either H&E staining or immunohistochemistry (IHC). Histopathological analysis was performed by a pathologist with expertise in human and murine malignancies (AKS).

Sections of tumor tissue for IHC staining were cut on SuperFrost Plus charged slides (Fisher). Unstained sections were deparaffinized, subjected to antigen retrieval and stained with antibody against SV40 TAg (Pab 101, 1:100) as described [27].

### Bioluminescent imaging (BLI)

For detection of *in vivo *growth of pWZL-Luc transduced MOVCAR tumor cells, mice were anesthetized with 2% isofluorane and given i.p. injections of 100 mg/kg luciferin substrate (Caliper Life Sciences) ten minutes prior to imaging using the IVIS Spectrum *in vivo *imaging system (Caliper Life Sciences) as described [49]. Image analysis was performed and total flux emission (photons/second) in the region of interest (ROI) was determined using the Living Image Software for the IVIS Spectrum.

## Results

### Allografted MOVCAR cells grow in immunodeficient mice, but not in wild type C57BL/6 mice

**Figure 1:**
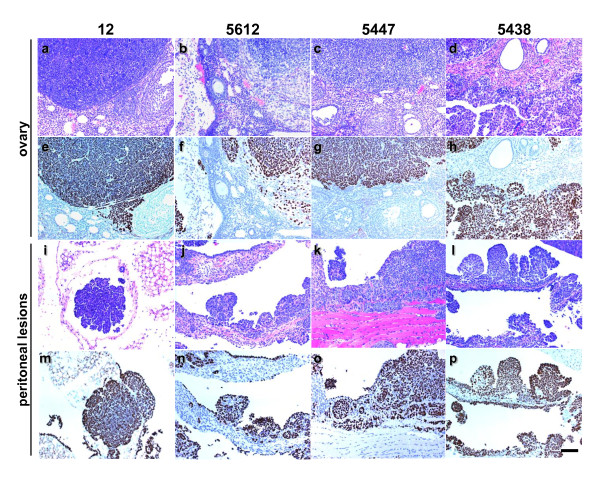
**Cell lines derived from C57BL/6 mice are tumorigenic in SCID mice**. Individual MOVCAR cell lines isolated from C57BL/6 mice (MOVCAR 12, 5612, 5447 and 5438) were tested for tumorigenicity in SCID mice by i.p. injection of 0.5 - 1.0 × 10^7 ^cells. H&E stained sections show the presence of tumor cells in the ovary (a-d) and peritoneum (i-l). The tumors derived from all cell lines were poorly differentiated carcinomas. The neoplastic cells were usually arranged in solid sheets and occasionally formed glandular structures and/or irregular slit-like spaces. On the peritoneal surface, these cells also formed papillary structures. Immunohistochemical detection of TAg (e-h and m-p) shows positively staining tumor cells with no staining of surrounding normal tissue. All micrographs were taken at the same magnification and the calibration bar shown in panel p corresponds to 100 μm.

Previous work showed that MOVCAR cell lines could be readily established from the malignant ascites of individual female Tg*MISIIR-TAg *founder mice with ovarian tumors and that these cells were tumorigenic in immunocompromised SCID mice [27]. Subsequently, MOVCAR cell lines have been isolated from the EOC-bearing female offspring of a fully penetrant stable transgenic line of EOC-prone mice, Tg*MISIIR-TAg*-DR26, derived from a male founder [28]. These cells exhibited the capacity for pseudo-orthotopic tumor growth giving rise to disseminated peritoneal tumors in SCID mice similar to advanced EOC observed in humans (data not shown). While the ability to grow tumor cells *in vivo *in immunodeficient animals is highly valuable for tumor biology studies, it is somewhat limited in that important contributions of immune cell signaling in the tumor microenvironment are lacking. Therefore, the ability to grow tumor cells in a syngeneic host is highly desirable. In establishing such a model, important considerations include the genetic background of both the host from which the tumor cells were isolated and the recipient animal into which they will be allografted. An additional consideration is the potential immunogenicity of the transgene protein product if it is not expressed endogenously in wild type mice, as is the case for SV40 TAg. All Tg*MISIIR-TAg *transgenic mice were initially established in a B6C3F1 first generation hybrid genetic background and maintained by crossing to wild type C57BL/6 mice, thus resulting in a mixed genetic background of the offspring and any cell lines derived from these mice. To address this issue, male Tg*MISIIR-TAg-DR6 *mice were maintained as hemizygotes with respect to the TAg transgene and backcrossed to wild type female C57BL/6 mice for a minimum of ten generations to ensure >99% purity of the C57BL/6 genetic background. No changes in either tumor latency or TAg expression patterns in ovarian tumors and reproductive tracts of female mice were observed during the process of backcrossing. Several new MOVCAR cell lines (MOVCAR 12, 5009, 5025, 5438, 5447 and 5612) were established from the ascites of ovarian tumor bearing pure C57BL/6 Tg*MISIIR-TAg-DR6 *mice and tested for tumorigenic potential following i.p. injection of 5 × 10^6 ^- 1 × 10^7 ^cells in SCID mice. Tumors developed within one to five months in SCID mice injected with all six cell lines tested (Figure 1, Table 1 and data not shown). In addition to the presence of peritoneal tumor nodules on the pancreas, omentum, mesentery, body wall and diaphragm, several of the SCID mice exhibited grossly enlarged ovaries at necropsy and histopathological review of H&E and TAg stained sections confirmed the presence of TAg positive tumor around and within the ovarian cortex. Tumors exhibited histology similar to high-grade serous ovarian carcinomas in women. Next, we similarly tested the tumorigenicity of MOVCAR cells in wild type C57BL/6 mice (n= 5 - 10 mice/group). Although each cell line tested was tumorigenic in SCID mice, none of the cell lines engrafted in immunocompetent wild type C57BL/6 mice (Table 1 and data not shown). The lack of tumor development in the immunocompetent C57BL/6 mice suggests, as previous studies have shown [50], that the expression of TAg proteins in the MOVCAR cells was immunogenic in wild type C57BL/6 recipients.

**Table 1:** Growth of MOVCAR cells in C57BL/6 and SCID mice

Host	MOVCAR cell line	**# cells injected i.p**.	Survival (days post tumor cell injection)	Tumor location	Ascites (>1.0 mL)
C57BL/6	12	1 × 10^7^	243	None	
C57BL/6	12	1 × 10^7^	256	None	
C57BL/6	12	1 × 10^7^	256	None	
C57BL/6	12	1 × 10^7^	256	None	
C57BL/6	12	2 × 10^7^	326	None	
C57BL/6	12	2 × 10^7^	326	None	
C57BL/6	12	3 × 10^7^	208	None	
C57BL/6	12	3 × 10^7^	208	None	
C57BL/6	12	3 × 10^7^	303	None	
C57BL/6	12	3 × 10^7^	303	None	
SCID	12	1 × 10^7^	93	Peritoneal cavity, invasion of ovarian cortex	+
SCID	12	1 × 10^7^	100	Peritoneal cavity, invasion of ovarian cortex	+
SCID	12	1 × 10^7^	103	Peritoneal cavity, invasion of ovarian cortex	+
SCID	12	1 × 10^7^	103	Peritoneal cavity, invasion of ovarian cortex	+
SCID	12	1 × 10^7^	105	Peritoneal cavity, invasion of ovarian cortex	+
SCID	5009	1 × 10^7^	25	Peritoneal cavity	+
SCID	5009	1 × 10^7^	25	Peritoneal cavity	+
SCID	5009	1 × 10^7^	34	Peritoneal cavity	+
SCID	5009	1 × 10^7^	34	Peritoneal cavity	+
SCID	5009	1 × 10^7^	34	Peritoneal cavity	+
SCID	5009	1 × 10^7^	34	Peritoneal cavity	+
SCID	5183	1 × 10^7^	109	Peritoneal cavity, invasion of ovarian cortex	+
SCID	5183	1 × 10^7^	116	Peritoneal cavity, invasion of ovarian cortex	
SCID	5183	1 × 10^7^	116	Peritoneal cavity, invasion of ovarian cortex	
SCID	5348	1 × 10^7^	141	Peritoneal cavity	+
SCID	5348	1 × 10^7^	141	Peritoneal cavity, invasion of ovarian cortex	+
SCID	5348	5 × 10^6^	141	Peritoneal cavity	+
SCID	5447	1 × 10^7^	95	Peritoneal cavity, invasion of ovarian cortex	+
SCID	5447	1 × 10^7^	95	Peritoneal cavity, invasion of ovarian cortex	+
SCID	5447	5 × 10^6^	95	Peritoneal cavity	
SCID	5447	5 × 10^6^	97	Peritoneal cavity, invasion of ovarian cortex	+
SCID	5612	1 × 10^7^	74	Peritoneal cavity, invasion of ovarian cortex	
SCID	5612	5 × 10^6^	97	Peritoneal cavity	+

### Analysis of SV40 TAg expression and function in MOVCAR cell lines

**Figure 2:**
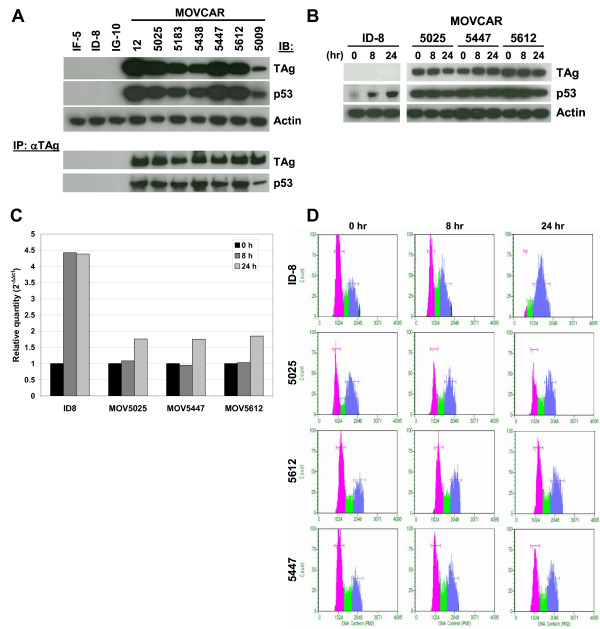
**Analysis of SV40 TAg expression and function in MOVCAR cells**. A) Whole cell lysates of spontaneously transformed MOSEC lines (IF-5, ID-8 and IG-10) and MOVCAR cell lines (12-3, 5025, 5183, 5438, 5447, 5612 and 5009) were evaluated by immunoblot analysis to determine relative levels of SV40 TAg, p53 and Actin (loading control) protein expression. Lysates were also immunoprecipitated with anti-TAg antibody Pab 101 followed by immunoblot analysis of TAg and p53 protein present in the immunoprecipitates. B) Induction of p53 protein was evaluated by immunoblot following treatment of ID-8 and MOVCAR 5025, 5447 and 5612 cells with 200 nM etoposide for 0, 8 and 24 hr. C) Levels of *Mdm2 *gene expression in ID-8 and MOVCAR 5025, 5447 and 5612 cells following treatment with 200 nM etoposide for 0, 8 and 24 hr were evaluated by qRT-PCR. D) Cell cycle analysis was performed on ID-8 and MOVCAR 5025, 5447 and 5612 cells following treatment with 200 nM etoposide.

One of the principle mechanisms of oncogenicity of SV40 virus is the capacity of the large TAg protein to bind to and functionally inactivate the p53 and Rb tumor suppressor proteins [51]. Expression of the large TAg protein was verified by Western blot in all of the MOVCAR cell lines, but absent in murine NIH3T3 cells (data not shown) or MOSEC cell lines IF-5, ID-8, and IG-10 (Figure 2A). In cells expressing wild type *p53*, p53 protein is kept at low, typically undetectable levels by ubiquitin mediated proteasomal degradation [52]. However, in cells expressing SV40 Large TAg, p53 protein remains bound to the TAg, resulting in p53 protein stabilization [52]. Consistent with these previous observations and our own published results showing p53 protein stabilization in Tg*MISIIR-TAg *ovarian tumors [27], we observed consistently high levels of p53 protein in MOVCAR cell lines, but not in MOSEC cell lines IF-5, ID-8, and IG-10 or NIH3T3 cells (Figure 2A and data not shown). Physical interaction of the TAg and p53 proteins in MOVCAR cells was confirmed by coimmunoprecipitation assay. Whole cell lysates immunoprecipitated with a TAg-specific antibody (Pab 101) and probed for p53 showed that p53 protein co-precipitated with TAg in all of the MOVCAR cells tested (Figure 2A, lower panels). To confirm that TAg binding results in the functional abrogation of p53, MOVCAR cells were treated with 200 nM etoposide for 0, 8 and 24 hours. The capacity for a p53-mediated response to etoposide treatment was assessed by evaluation of p53 protein expression and stabilization, induction of the p53 responsive gene *Mdm2 *and induction of cell cycle arrest. Treatment of the TAg negative ID-8 cells with etoposide resulted in induction and stabilization of p53 protein (Figure 2B), suggesting that p53 is functional in these cells. However, in TAg expressing MOVCAR cells, p53 protein was already stabilized and no further induction or stabilization of p53 was observed in the etoposide treated compared to untreated cells (Figure 2B). In etoposide treated ID-8 cells, qRT-PCR analysis showed greater than four-fold induction of *Mdm2 *expression (Figure 2C) and cell cyle analysis showed growth arrest indicated by accumulation of cells in G2/M (Figure 2D). None of the similarly treated TAg positive MOVCAR cell lines exhibited robust induction *Mdm2 *expression or G2/M growth arrest. Taken together, these results confirm the functional activity of TAg in MOVCAR cell lines.

### VEGF secretion in MOVCAR cell lines

In culture, MOVCAR cells exhibit differences in growth rates and expression of signaling proteins associated with EOC, including VEGF among others (Additional file 1, Table S1 and data not shown). Differences in tumor growth rates and ascites production among different MOVCAR cell lines were also apparent *in vivo*. Peritoneal implantation of MOVCAR 5009 or 5025 cells in SCID mice resulted in rapid tumor growth and the production of voluminous ascites that necessitated euthanasia within 4-6 weeks. In SCID mice injected with MOVCAR 5183, 5438, 5447 and 5612 cells, the time to development of tumors necessitating euthanasia was between 12 and 20 weeks and mice generally exhibited lower volumes of ascites at the time of necropsy (Table 1 and data not shown). The cell lines expressing the highest levels of secreted VEGF *in vitro *(e.g., MOVCAR 5009 and 5025) resulted in more rapid tumor growth and ascites production *in vivo *than cell lines with lower VEGF levels. This observation is consistent with a previous study showing that enforced expression of VEGF in the spontaneously transformed MOSEC line ID-8 led to more aggressive *in vivo *tumor growth and more ascites production than the parental cell line [18]. Like individual MOSEC lines [10], the results also suggest that although MOVCAR cell lines are derived from ascites from an inbred strain of transgenic mice, individual cell lines exhibit intrinsic differences.

### Oophorectomized C57BL/6 TgMISIIR-TAg-DR6 mice develop intrabursal and disseminated peritoneal carcinomas

**Figure 3:**
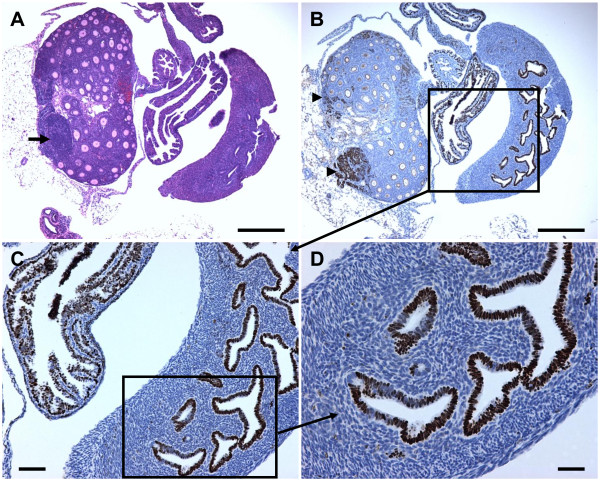
**Early tumor formation in Tg*MISIIR-TAg-DR26 *mice**. The presence and extent of tumor formation in a 28-day old female Tg*MISIIR-TAg-DR26 *mouse was confirmed by histopathological evaluation. A) Low power magnification (40x) of an H&E stained section of the reproductive tract showing the ovary and segments of the fallopian tube and uterus reveals an early-stage ovarian tumor indicated by the arrow. B) Immunostaining of an adjacent section shows TAg positive tumor cells in the ovary (arrowheads). TAg positive staining cells were also apparent in the epithelium of the fallopian tube and endometrium. The segment contained within the box is shown in (C) at higher magnification (100X). D) High power magnification (400X) of the boxed area in (C) showing the TAg positive epithelial cells of the endometrial glands. *Calibration bar*: A and B, 1 mm; C, 250 μm; D, 125 μm.

In order to identify a suitable syngeneic recipient strain for *in vivo *growth, one potential strategy to overcome immunogenicity of the TAg transgene proteins is to grow MOVCAR cells in tumor-prone C57BL/6 Tg*MISIIR-TAg-DR6 *transgenic mice. We hypothesized that removal of the ovaries of young Tg*MISIIR-TAg-DR26 *transgenic mice might abrogate tumor development and render these mice suitable for engraftment of MOVCAR cells. In addition to TAg expression detected in tumor cells, TAg staining was also commonly observed in the uterine and fallopian tube epithelia of 28 day-old mice (Figure 3 and [28]), although neither uterine nor fallopian tube carcinomas were observed at the time of euthanasia. However, it is possible that ovarian carcinoma development was sufficiently rapid that it outpaced carcinoma development in the endometrium or oviduct. To determine whether removal of the ovaries from Tg*MISIIR-TAg-DR26 *transgenic mice was sufficient to inhibit tumor formation, a series of oophorectomy experiments were performed (summarized in Table 2). Mice were oophorectomized between four and six weeks of age, which is prior to the age of onset of cyclivity at 48 days in C57BL/6 mice [53] and prior to any obvious enlargement of the ovaries (Figure 3, [28] and data not shown). Female C57BL/6 Tg*MISIIR-TAg-DR26 *transgenic mice were subjected to the following surgical manipulations: 1) bilateral oophorectomy (n = 9), 2) bilateral oophorectomy and salpingectomy (n = 5) and 3) bilateral oophorectomy and salpingectomy with removal of the ovarian bursa (n = 8) and the results are summarized in Table 2. Tumor formation was detected in most mice and histopathological evaluation revealed the presence of carcinomas that were similar to those that occurred spontaneously in Tg*MISIIR-TAg-DR26 *transgenic mice. Tumors arising in the Tg*MISIIR-TAg-DR26 *mice in which the ovarian bursa was removed at the time of bilateral oophorectomy and salpingectomy were widely disseminated in the peritoneal cavity and resembled primary peritoneal carcinomatosis. The origin of the tumors remains uncertain as 28 day-old mice already exhibited the presence of TAg positive tumor cells in the ovary and TAg positive cells in the fallopian tube and uterus (Figure 3 and [28]). Tumors arising in ovariectomized mice may originate from residual tumor cells shed from the ovaries prior to the time of surgery, or alternatively, from TAg positive cells present in the fallopian tubes or the uterus. Although we cannot definitively distinguish between these possibilities, the histology of tumors arising in oophorectomized mice resembled the high-grade serous ovarian adenocarcinomas and disseminated peritoneal carcinomatosis that occurs spontaneously in Tg*MISIIR-TAg-DR26 *mice suggesting that ovarian tumors arise from the ovaries and/or fallopian tube and that tumor initiation occurs early in these mice. There was no evidence of endometrial carcinomas in any of the groups, suggesting that although the SV40 TAg transgene protein is expressed in the endometrium, this expression is not sufficient for full oncogenic transformation of this tissue. Importantly, as surgical removal of the ovaries and oviduct are not sufficient to prevent tumor development, these mice are unsuitable as allograft hosts for implantation of MOVCAR cells.

**Table 2:** Oophorectomized Tg*MISIIR-TAg *transgenic mice develop epithelial tumors.

Surgical procedure	Number of mice with tumors
1) Remove TgMISIIR-TAg ovaries	9/9
2) Remove TgMISIIR-TAg ovaries and fallopian tubes	4/5
3) Remove TgMISIIR-TAg ovaries, fallopian tubes and bursa	6/8

### Phenotypes of TgMISIIR-TAg transgenic mice

**Figure 4:**
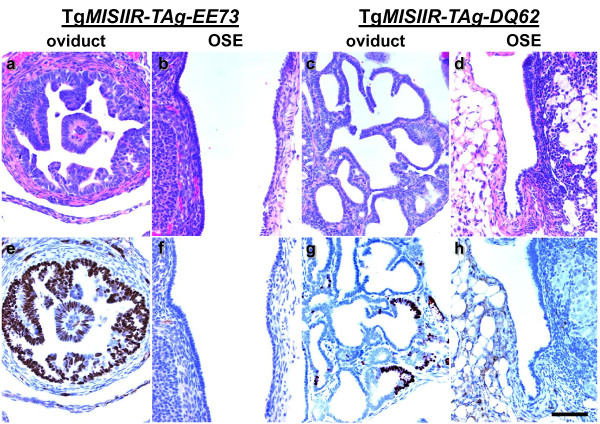
**Tg*MISIIR-TAg-EE73 *and Tg*MISIIR-TAg-DQ62 *mice exhibit restricted TAg expression**. Histopathological evaluation of H&E (a-d) and TAg (e-h) stained sections of female Tg*MISIIR-TAg-EE73 *(a, b, e and f) and Tg*MISIIR-TAg-DQ62 *(c,d, g and h) mice show TAg positive cells present in the oviduct (e and g), but not in the OSE and bursal epithelium of the same mice (f and h). All micrographs were taken at the same magnification and the calibration bar shown in panel h corresponds to 100 μm.

As an alternative means to circumvent the problem of TAg immunogenicity in recipient mice, we used a strategy previously described by Mintz and Silvers [54] in which inbred transgenic mice with low expression of the tumor promoting transgene, and hence little or no susceptibility to tumor formation, were utilized as allograft recipients. To identify such transgenic lines, we isolated and phenotypically analyzed a total of 96 TAg positive Tg*MISIIR-*TAg transgenic founders. Among these, 36 were female, and as previously reported [27], 18/36 (50%) developed early onset, bilateral, moderately to poorly differentiated ovarian carcinomas with widespread peritoneal dissemination. Tumors exhibiting differentiated morphology resembled high-grade serous EOCs. Among the remaining female founders, 3/36 (8%) developed non-ovarian tumors and 15/36 (42%) lacked detectable TAg transgene protein expression, failed to transmit the transgene or failed to breed. Therefore, TAg positive male founders were bred and offspring were analyzed to identify stable transgenic lines of mice that transmitted the TAg transgene. Similar to female Tg*MISIIR-TAg *founder mice, male founders were frequently infertile, sub-fertile or did not transmit transgene expression. Among the fertile transgenic lines established from Tg*MISIIR-TAg *male founders, several exhibited TAg transgene expression in the fallopian tubes of female offspring without obvious pathology. Further phenotypic characterization of female offspring of two of these transgenic lines, Tg*MISIIR-TAg-DQ62 *and Tg*MISIIR-TAg-EE73*, showed that although the mice expressed the TAg transgene, they exhibited normal fertility and lifespan and failed to develop tumors. The expression of TAg protein in these mice was detected in a limited number of epithelial cells lining the fallopian tube (Figure 4). These transgenic lines are referred to as "Tg*MISIIR-TAg*-*Low*" mice due to the relatively limited expression of TAg protein. Previous work [54] suggested that because these mice normally expressed the TAg protein and exhibited little or no susceptibility tumor formation, they could serve as suitable hosts for implantation of TAg expressing MOVCAR cells.

### MOVCAR cells grow as i.p. and orthotopic allografts in C57BL/6 TgMISIIR-TAg-Low recipients

**Figure 5:**
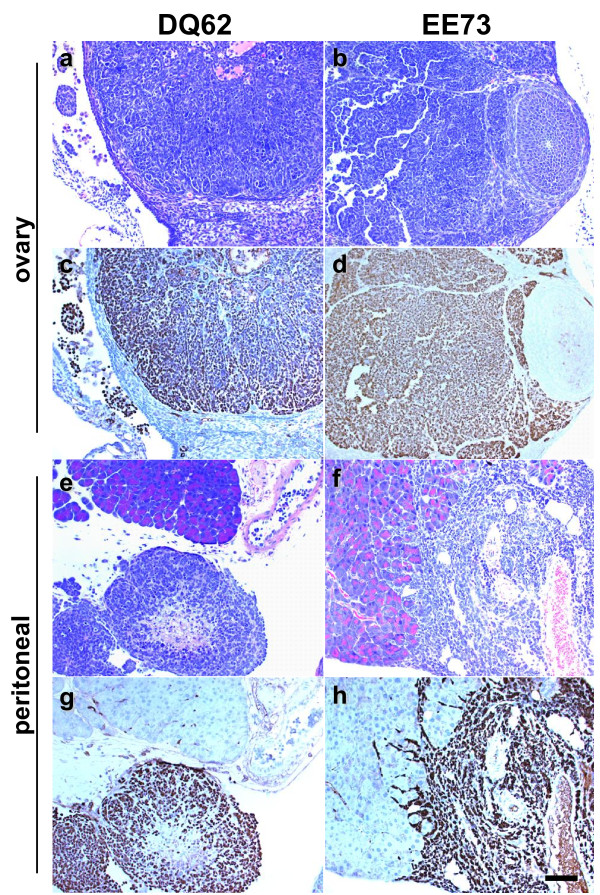
**MOVCAR 12 cells are tumorigenic in Tg*MISIIR-TAg-DQ62 *and Tg*MISIIR-TAg-EE73 *mice**. Micrographs of tumors arising in Tg*MISIIR-TAg-DQ62 *(a, c, e and h) and Tg*MISIIR-TAg-EE7 *(b, d, f and h) mice injected i.p. with 2 × 10^7 ^MOVCAR 12 cells. H&E (a, b, e and f) and TAg (c, d, g and h) stained sections of tumors located within the ovarian cortex (a-d) and peritoneal tumors invading the omentum and pancreas (e-h). The tumors were similar to those described in Figure 1. All micrographs were taken at the same magnification and the calibration bar shown in panel h corresponds to 100 μm.

**Figure 6:**
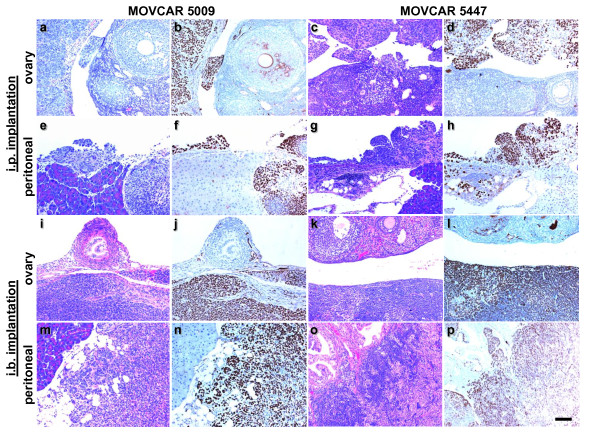
**Pseudo-orthotopic and orthotopic implantation of MOVCAR cell in Tg*MISIIR-TAg-Low *mice**. H&E (a, c, e, g, I, k, m and o) and TAg (b, d, f, h, j, l, n and p) stained sections of tumors arising in Tg*MISIIR-TAg-Low *mice injected i.p (a-h) or i.b. (i-p) with MOVCAR 5009 (a, b, e, f, i, j, m and n) and 5447 (c, d, g, h, k, l, o and p) cells. The tumors are similar to those shown in the previous figures, i.e., poorly differentiated carcinomas with few areas of tubular differentiation and occasional papillary structures, e.g., panels c and g. All micrographs were taken at the same magnification and the calibration bar shown in panel p corresponds to 100 μm.

Prior to testing whether the Tg*MISIIR-TAg-Low *transgenic lines, DQ62 and EE73, could serve as recipients for allografted MOVCAR cells, each was backcrossed to wild type C57BL/6 mice for a minimum of ten generations to ensure genetic purity. No changes in TAg expression patterns in the reproductive tracts of female mice were observed during the backcrossing process. To test whether MOVCAR cells could be grown as allografts in female C57BL/6 Tg*MISIIR-TAg-Low *transgenic mice, three Tg*MISIIR-TAg-DQ62 *mice and three Tg*MISIIR-TAg-EE73 *mice were each injected i.p. with 2 × 10^7 ^MOVCAR 12 cells. Similar to SCID mice, C57BL/6 Tg*MISIIR-TAg-DQ62 *and Tg*MISIIR-TAg-EE73 *mice injected i.p. with MOVCAR 12 cells developed tumors that necessitated euthanasia within three months (Figure 5 and Table 3). At necropsy, disseminated peritoneal tumors were detected and several mice exhibited enlarged ovaries. In addition to the presence of disseminated peritoneal adenocarcinoma infiltrating the pancreas, omentum, mesentery, diaphragm and abdominal wall, histopathological review of H&E and TAg stained sections revealed the presence of tumor cells growing within the intrabursal space surrounding the ovaries and within the ovarian cortex of both the C57BL/6 Tg*MISIIR-TAg-DQ62 *and Tg*MISIIR-TAg-EE73 *mice (Figure 5 and Table 3). Detection of the TAg positive tumor cells in the ovaries of both SCID (Figure 1) and syngeneic C57BL/6 Tg*MISIIR-TAg-Low *mice (Figure 5) suggests that MOVCAR cells exhibit a strong propensity for organotropic homing to ovary. To ensure that the observed results were not cell line-specific, five additional MOVCAR cell lines (MOVCAR 5009, 5025, 5183, 5447 and 5612) were tested for tumorigenic potential following i.p. and i.b. injection. All five cell lines tested grew as allografts in C57BL/6 Tg*MISIIR-TAg-Low *mice (Figure 6, Table 3 and data not shown) producing disseminated peritoneal adenocarcinoma frequently accompanied by intrabursal and intra-ovarian tumor growth. Like the allograft experiments performed in SCID mice, individual cell lines exhibited differences in tumor latency and dissemination pattern in C57BL/6 Tg*MISIIR-TAg-Low *mice. However, the tumor latency and dissemination pattern for any individual cell line are similar in SCID and C57BL/6 Tg*MISIIR-TAg-Low *allograft recipients (compare data summarized in Tables 1 and 3). Taken together, these results show that both lines of C57BL/6 Tg*MISIIR-TAg-Low *mice can serve as immunocompetent syngeneic recipients for the growth of MOVCAR tumor cells isolated from individual tumor bearing C57BL/6 Tg*MISIIR-TAg*-*DR6 *mice.

**Table 3:** Growth of MOVCAR cells in Tg*MISIIR-TAg-Low *mice

Host	MOVCAR cell line	**# cells injected i.p**.	Survival (days post tumor cell injection)	Tumor location	Ascites (>1.0 mL)
DQ62	12	2 × 10^7^	96	Peritoneal cavity, invasion of ovarian cortex	+
DQ62	12	2 × 10^7^	90	Peritoneal cavity, invasion of ovarian cortex	+
DQ62	12	2 × 10^7^	96	Peritoneal cavity, invasion of ovarian cortex	+
EE73	12	2 × 10^7^	90	Peritoneal cavity, invasion of ovarian cortex	
EE73	12	2 × 10^7^	96	Peritoneal cavity, invasion of ovarian cortex	
EE73	12	2 × 10^7^	90	Peritoneal cavity, invasion of ovarian cortex	
DQ62	5009	5 × 10^6^	28	Peritoneal cavity	+
DQ62	5009	5 × 10^6^	28	Peritoneal cavity	+
DQ62	5009	5 × 10^6^	28	Peritoneal cavity	+
DQ62	5009	5 × 10^6^	28	Peritoneal cavity	+
DQ62	5025	5 × 10^6^	30	Peritoneal cavity, invasion of ovarian cortex	+
DQ62	5025	1 × 10^7^	30	Peritoneal cavity, invasion of ovarian cortex	+
DQ62	5025	1 × 10^7^	37	Peritoneal cavity, invasion of ovarian cortex	+
DQ62	5025	1 × 10^7^	37	Peritoneal cavity, invasion of ovarian cortex	+
DQ62	5183	2 × 10^7^	108	Peritoneal cavity	
DQ62	5183	2 × 10^7^	49	Peritoneal cavity	+
DQ62	5612	1.5 × 10^7^	71	Peritoneal cavity	+
DQ62	5612	1.5 × 10^7^	71	Peritoneal cavity	+

### Tumor growth in TgMISIIR-TAg-Low mice can be monitored in vivo by bioluminescent imaging

Although orthotopic or pseudo-orthotopic implantation of EOC cells represents a more highly relevant tumor microenvironment for tumor growth, there are inherent difficulties in detection and quantitation of tumor growth and progression in deeply embedded tumors growing within the intrabursal space or as disseminated peritoneal disease. To facilitate detection and quantitation of tumor growth *in vivo*, MOVCAR 5009 and 5447 cells were transduced with a retroviral construct encoding firefly luciferase. Stably transduced cells were implanted into C57BL/6 Tg*MISIIR-TAg-Low *mice by i.p. or i.b. injection and tumor growth was then monitored non-invasively by bioluminescent imaging (BLI).

**Figure 7:**
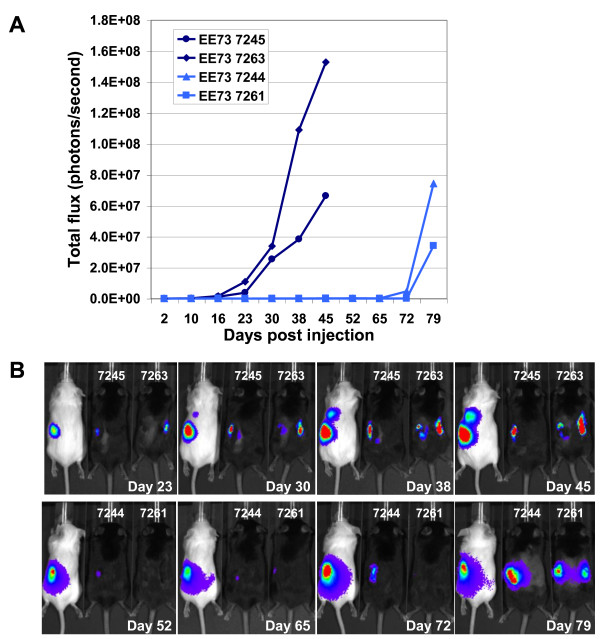
**Orthotopic tumor growth in Tg*MISIIR-TAg-Low *mice monitored and quantified *in vivo *by BLI**. SCID and Tg*MISIIR-TAg-EE7 *mice were given unilateral or bilateral intrabursal injections with 2 × 10^5 ^MOVCAR 5009 or 5447 cells and subjected to weekly bioluminescent imaging to monitor tumor growth. A) Quantitative analysis of total photon counts from dorsal images of Tg*MISIIR-TAg-EE7 *mice injected i.b. with MOVCAR 5009 cells (mice 7245 and 7263) and MOVCAR 5447 cells (mice 7244 and 7261). B) Dorsal images of control SCID and Tg*MISIIR-TAg-EE7 *mice injected i.b. with MOVCAR 5009 cells (mice 7245 and 7263) and MOVCAR 5447 cells (mice 7244 and 7261) showing proscribed luminescent signals at the site of unilateral (mice 7245 and 7244) or bilateral (mice 7263 and 7261) i.b. injection.

Age matched female C57BL/6 Tg*MISIIR-TAg-Low *(DQ62) mice injected i.p. with 5.0 × 10^6 ^MOVCAR-5009-Luc cells rapidly developed disseminated peritoneal carcinomatosis readily detectable by BLI (data not shown). We predicted that detection of bioluminescent signal emanating from orthotopically implanted tumors would be technically more challenging in C57BL/6 Tg*MISIIR-TAg-Low *mice due to the relatively low number of cells that can be implanted by this method (e.g., 8.0 × 10^5 ^cells) and by the presence of black pigment in the C57BL/6 mice compared to white mice. Therefore, SCID mice injected i.b. with the same number of either MOVCAR-5009-Luc or MOVCAR-5447-Luc cells were used as positive controls for BLI of orthotopical	ly implanted tumor cells in age-matched female C57BL/6 Tg*MISIIR-TAg-Low *mice injected i.b. with 8.0 × 10^5 ^MOVCAR-5009-Luc (mouse numbers EE73 7245 and EE73 7263) or MOVCAR-5447-Luc (mouse numbers EE73 7244 and EE73 7261) cells (Figure 7). Tumor growth was monitored by BLI for up to 11 weeks and showed that orthotopic implantation of cells resulted in proscribed luminescent signals that were confined to the site of intrabursal injection in both SCID and C57BL/6 Tg*MISIIR-TAg-Low *mice (Figure 7 and Table 4). Although the same numbers of cells were injected in all mice, signal intensities appear stronger in SCID mice due to the lack of pigment and therefore, are not directly comparable to BLI signals in the C57BL/6 Tg*MISIIR-TAg-Low *mice. *In vitro*, MOVCAR-5009-Luc cells grow much more rapidly than MOVCAR-5447-Luc cells. This pattern was also observed *in vivo*, with rapid acceleration of tumor growth detected three weeks post injection of MOVCAR-5009-Luc cells, while signal intensities detected in mice injected with MOVCAR-5447-Luc cells did not increase significantly until ten weeks post injection (Figure 7). Taken together, these data show that growth of MOVCAR cells engineered to express firefly luciferase can be monitored non-invasively by BLI and that differences in *in vivo *growth rates of individual MOVCAR cell lines can be detected using this method.

**Table 4:** Intrabursal growth of MOVCAR-Luciferase cells in Tg*MISIIR-TAg-Low *mice

Host	MOVCAR cell line	**# cells injected i.b**.	site of injection	Survival (days post tumor cell injection)	Right ovary tumor volume **(mm**^**3**^**)**	Left ovary tumor volume **(mm**^**3**^**)**	Ascites (>1.0 mL)
EE73	5009	8 × 10^5^	left	50	n/a	167	+
EE73	5009	8 × 10^5^	bilateral	50	151	176	+
EE73	5447	8 × 10^5^	left	81	n/a	57	
EE73	5447	8 × 10^5^	bilateral	81	32	76	

n/a: not applicable

## Discussion

Utilization of animal models with an intact immune system is critical for the evaluation of immune-based therapeutic strategies and vaccine development. An SV40 TAg transgenic model of prostate cancer [55] has been used to study the effects of combining blockade of cytotoxic T lymphocyte antigen 4 (CTLA-4) and vaccination with granulocyte macrophage colony stimulating factor (GM-CSF;Gvax) and subsequent derivatives of this vaccine strategy [56-60]. The C57BL/6 syngeneic mouse ovarian cancer model developed by Roby et al, [10] has been used for studies of the contribution of cells in the tumor microenvironment, including epithelial-stromal cell interactions, VEGF induced-effects on tumor vasculature and tumor cell-secreted factors that stimulate cytokine production, macrophage infiltration and vascularization that favor tumor growth and progression [14,15,18]. Similar studies would be difficult to impossible to conduct in immunodeficient mice. The availability of an additional syngeneic mouse model of EOC will allow cross-comparison of mouse models and validation of key findings.

The functional utility of animal models of human cancer depends largely on the extent to which the animal model recapitulates the histology and biological behavior of the disease in humans. Many transgenic tumor models have been developed using the immediate early region of the SV40 virus containing the potently oncogenic large and small T antigen (*TAg *and *tag*) genes [55,61-66]. The continued utility of SV40 TAg models in studying cancer is underscored by seminal contributions to our understanding of the "angiogenic switch" [67-71] and tumor progression and invasion [72]. Importantly, a recent study [73] identified an integrated gene expression signature from three distinct TAg mouse models (i.e., mammary, prostate and lung cancer models) that is comparable to a signature associated with the aggressive biological behavior and prognosis for several human epithelial tumors, including breast cancers. Results from this study showed that tumors arising in TAg-based mouse models share common features of gene expression with human cancer and are relevant preclinical models [73].

Female transgenic C57BL/6 Tg*MISIIR-TAg-DR26 *mice develop spontaneous bilateral ovarian carcinoma with 100% penetrance [28]. Tumor progression in these mice is characterized by widespread peritoneal dissemination and the development of malignant ascites and tumor morphology and histology of the tumors closely resembles high-grade serous adenocarcinomas, the most common histologic subtype of EOC detected in women. Tumors and cell lines derived from primary tumors and ascites of tumor bearing mice exhibit several characteristics in common with human EOC cell lines and tumors including AKT/mTOR activation, COX1 overexpression and VEGF overexpression and secretion ([28,44-47] and the present study). In addition, a verapimil-sensitive Hoescht dye-excluding ovarian carcinoma side population (SP), a potential population of ovarian cancer initiating cells, was identified in MOVCAR cell lines [48]. Ovarian tumors arising in C57BL/6 Tg*MISIIR-TAg-DR26 *mice are sensitive to standard combination platinum and taxane chemotherapy and to mTOR inhibition with Everolimus (RAD001) [28,45]. These observations underscore the potential utility of these transgenic mice for preclinical evaluation of therapeutic agents. However, reflecting its relation to the biology of human EOC, tumor formation in this transgenic model is also stochastic, resulting in variation in the latency of tumor formation and time to metastasis. This necessitates relatively large cohorts of mice and non-invasive longitudinal *in vivo *imaging such as MRI to optimize results of therapeutics studies.

To overcome the limitations encountered with spontaneous tumor development, we isolated individual transgenic lines of non-tumor prone C57BL/6 Tg*MISIIR-TAg *transgenic mice that can serve as syngeneic immunocompetent hosts for allografted TAg expressing MOVCAR cells isolated from tumor bearing C57BL/6 Tg*MISIIR-TAg-DR26 *mice. Syngeneic mouse models of EOC in which spontaneously transformed ID-8 MOSEC grown as allografts in C57BL/6 recipients [10] or HM-1 tumor cells grown as allografts in B6C3F1 recipients [74] have been previously described. These syngeneic models have been used successfully for preclinical evaluation of therapeutic agents and studies of the role of the tumor microenvironment on ovarian tumor growth and progression [11-18,75]; however, these models each rely on single mouse ovarian carcinoma cell lines in which the underlying molecular mechanisms of malignant transformation remain undefined.

The ease of establishment of TAg-transformed MOVCAR cell lines in culture has enabled the isolation of a large number of distinct cell lines, several of which are described in the present study. Although derived from an inbred strain of mice, the stochastic manner in which tumors arise in C57BL/6 Tg*MISIIR-TAg-DR26 *mice results in intrinsic differences in MOVCAR cell lines derived from individual tumor-bearing mice. MOVCAR cell lines grown in culture exhibit different growth rates and expression of proteins associated with EOC, such as levels of secreted VEGF. These cell lines also exhibit differences when grown *in vivo*. For example, some cells lead to very rapid growth and production of voluminous malignant ascites, whereas other cells are slower growing and produce less ascites. Interestingly, the cell lines that result in the highest levels of ascites production *in vivo *are the cell lines that exhibit the highest levels of VEGF secretion *in vitro*. These observations suggest that although the primary oncogenic stimulus driving tumorigenesis in C57BL/6 Tg*MISIIR-TAg-DR26 *transgenic mice is the same in all animals, there are likely additional genetic, epigenetic and/or gene expression alterations that contribute to ovarian tumor progression, and identification of these alterations may contribute to our understanding of human EOC. Moreover, once identified, the role of specific alterations in gene function in ovarian tumorigenesis can be studied in these cell lines as they are readily amenable to direct manipulation using established strategies for ectopic gene expression or RNA interference.

With regard to preclinical evaluation of novel therapeutic agents, our syngeneic mouse model of EOC provides several advantages. First, tumors are grown in fully immunocompetent mice enabling the evaluation of vaccine and immune-based therapeutic strategies. Second, Tg*MISIIR-TAg-Low *transgenic mice have been fully backcrossed to a pure C57BL/6 genetic background, exhibit normal fertility and lifespan and do not develop tumors. Thus, large cohorts of mice can be established for synchronous allograft initiation without interference of tumor growth initiated from the host. Third, the availability of multiple distinct MOVCAR cells lines for evaluation avoids issues of cell line-specific effects, and because MOVCAR cells are easily manipulated in culture, on-target effects of therapeutics can be confirmed in parallel using RNAi based strategies for direct target knockdown. Finally, the ability to easily express reporter genes in MOVCAR cells facilitates strategies for non-invasive *in vivo *optical imaging such as bioluminescent, fluorescent and near infrared fluorescent imaging.

## Conclusions

In conclusion, we have developed an immunocompetent syngeneic mouse model of EOC consisting of C57BL/6 Tg*MISIIR-TAg-Low *transgenic mice that can serve as immunocompetent syngeneic allograft recipients for MOVCAR cell lines. Based on distinct characteristics of these cell lines and their amenability to *in vitro *manipulation of gene expression, this model represents a flexible system to study ovarian tumor biology and to evaluate the efficacy of novel therapeutic strategies.

## List of abbreviations

EOC: epithelial ovarian cancer; TAg: T antigen; SCID: severe combined immunodeficient; MOVCAR: murine ovarian carcinoma; OSE: ovarian surface epithelium; GEM: genetically engineered mouse; MISIIR: Müllerian inhibiting substance type II receptor; IHC: immunohistochemistry; BLI: bioluminescent imaging.

## Competing interests

The authors declare that they have no competing interests.

## Authors' contributions

BAQ, FX, LB and LM conducted the studies and participated in the data analysis. XH performed oophorectomies, ovarian transplants and orthotopic implantation of tumor cells and AKS conducted the histopathological evaluation of tumor tissues. DCC conceived and designed experiments, analyzed the data and wrote the manuscript. All authors have read and approved the final manuscript.

## Supplementary Material

Additional file 1 **Levels of secreted VEGF protein in MOVCAR cells**. The amount of secreted VEGF protein present in conditioned medium of seven independent MOVCAR cell lines was determined by ELISA assay. Click here for file
