# Supplementary material for: Development of a syngeneic mouse model of epithelial ovarian cancer
Source: J Ovarian Res. 2010 Oct 19;3:24. doi: 10.1186/1757-2215-3-24 (PMC2974672; doi:10.1186/1757-2215-3-24)
Supplement: Additional file 1 — Levels of secreted VEGF protein in MOVCAR cells. The amount of secreted VEGF protein present in conditioned medium of seven independent MOVCAR cell lines was determined by ELISA assay. [file 1757-2215-3-24-S1.DOC]

**A**

**B**

**Additional file 1. Levels of secreted VEGF protein in MOVCAR cells.** A) Levels of secreted VEGF present in conditioned culture medium of ID-8, ID-8 VEGF MOSEC and MOVCAR 12, 5009, 5025, 5183, 5438, 5447, 5612 cells were detected by ELISA. Equal numbers of cells were plated in triplicate, grown for 48 h and culture supernatant collected and subjected to ELISA. Cells were counted and the levels of secreted VEGF represented as pg/10^4^ cells. Assays were performed three times with data from replicate assays shown (error bars indicate SEM). B) Same as in A, excluding value for ID-8 VEGF cells.
